# The impact of lipid-based nutrient supplementation on anti-malarial antibodies in pregnant women in a randomized controlled trial

**DOI:** 10.1186/s12936-015-0707-2

**Published:** 2015-05-10

**Authors:** Upeksha P Chandrasiri, Freya JI Fowkes, Jack S Richards, Christine Langer, Yue-Mei Fan, Steve M Taylor, James G Beeson, Kathryn G Dewey, Kenneth Maleta, Per Ashorn, Stephen J Rogerson

**Affiliations:** Department of Medicine, Clinical Sciences Building, The University of Melbourne, Royal Parade, Parkville, VIC 3052 Australia; Macfarlane Burnet Institute of Medical Research, 85 Commercial Road, Melbourne, Australia; Centre for Epidemiology and Biostatistics, Department of Epidemiology, The University of Melbourne, Melbourne, Australia; Preventive Medicine and Department of Infectious Diseases, Monash University, Melbourne, Australia; Department for International Health, University of Tampere School of Medicine, Tampere, Finland; Department of Paediatrics, Tampere University Hospital, Tampere, Finland; Duke University Medical Center, Durham, North Carolina USA; Gillings School of Global Public Health, University of North Carolina, Chapel Hill, USA; Department of Nutrition, University of California Davis, Davis, California USA; College of Medicine, University of Malawi, Blantyre, Malawi

**Keywords:** Malaria in pregnancy, Malawi, nutrient supplements, placental-binding parasite isolate, opsonizing antibodies, immunoglobulin G isotypes, variant surface antigens, merozoite antigens, body mass index, socioeconomic status

## Abstract

**Background:**

Malaria and undernutrition frequently coexist, especially in pregnant women and young children. Nutrient supplementation of these vulnerable groups might reduce their susceptibility to malaria by improving immunity.

**Methods:**

Antibody immunity to antigens expressed by a placental-binding parasite isolate, a non-placental binding parasite isolate, merozoites and schizonts at enrolment (before 20 gestation weeks) and at 36 gestation weeks were measured in 1,009 Malawian pregnant women receiving a daily lipid-based nutrient supplement, multiple micronutrients or iron and folic acid, who were participants in a randomized clinical trial assessing the effects of nutrient supplementation on pregnancy outcomes and child development(registration ID: NCT01239693).

**Results:**

Antibodies to placental-binding isolates significantly increased while antibodies to most merozoite antigens declined over pregnancy. Overall, after adjustment for covariates, the type of supplementation did not influence antibody levels at 36 gestation weeks or the rate of change in antibody levels from enrolment to 36 weeks. A negative association between maternal body mass index and opsonizing antibodies to placental-binding antigens (coefficient (95% CI) -1.04 (−1.84, −0.24), was observed. Similarly, women with higher socioeconomic status had significantly lower IgG and opsonizing antibodies to placental-binding antigens. Neither of these associations was significantly influenced by the supplementation type.

**Conclusions:**

In the current cohort nutrient supplementation did not affect anti-malarial antibody responses, but poor and undernourished mothers should be a priority group in future trials.

**Electronic supplementary material:**

The online version of this article (doi:10.1186/s12936-015-0707-2) contains supplementary material, which is available to authorized users.

## Background

It is estimated that about 125 million pregnancies worldwide are at risk of malaria annually, significantly increasing maternal and child morbidity and mortality [1]. Undernutrition is prevalent in regions where women are at high risk of malaria in pregnancy [2]. This coexistence of undernutrition and malaria markedly increases the risk of adverse birth outcomes, such as intrauterine growth restriction, compared to malaria or undernutrition alone [3,4]. The complex relationship between malaria and nutrition has been studied [5,6], but understanding of its mechanisms remains limited. Malaria in pregnancy predisposes women to anaemia, and may result in reduced nutrient intake due to febrile illness and anorexia. Malaria also increases susceptibility to other infections through its immunosuppressive effects [7-9], whilst on the other hand both macronutrient and micronutrient deficiencies may increase the risk of infections including malaria [6]. This increased susceptibility is believed to be due to impairment of host immune defences [10] such as abnormalities in complement activation [11] and impaired cell mediated immunity [12] leading to reduced antibody production.

In endemic regions, pregnant women naturally acquire antibodies to malaria with repeated exposure to infectious mosquito bites prior to and during pregnancy. During first pregnancies, women frequently acquire antibodies to the pregnancy-specific antigens, primarily to VAR2CSA of *Plasmodium falciparum* erythrocyte membrane protein-1 family of variant surface antigens (VSA). These antibodies help protect women against adverse clinical outcomes in subsequent pregnancies [13]. However, undernourished women may have difficulty effectively maintaining or acquiring antibodies against malaria antigens.

In non-malaria-related studies both macro- and micronutrient supplementation have been shown to significantly improve pregnancy outcomes and maternal health [14,15]. Lipid-based nutrient supplements (LNS) are multiple micronutrient-fortified lipid-rich products that can be beneficial as prenatal supplements in vulnerable groups [16-18]. Prenatal LNS has been shown to improve birth length [16] and reduce weight loss in HIV-infected mothers [17], and in young HIV-exposed infants LNS is suitable as a breast milk replacement [19]. In addition, LNS may improve linear growth outcomes in young children [20,21].

The only study to date that has assessed the effects of maternal nutrient supplementation on malaria antibody levels reported that vitamin A prenatal supplements were associated with a reduction in antibody responses to a placental-binding isolate EJ-24, but no significant changes in antibody responses to non-pregnancy related parasite isolates were observed [22].

In areas with food insecurity and high malaria transmission, nutritional supplements could improve pregnancy outcomes and may also lead to stronger acquired immune responses to malaria. To investigate this, antibody immunity was measured to antigens expressed by placental-binding and non-placental-binding parasite isolates, merozoite antigens and schizont extract in pregnant women from Mangochi, Malawi enrolled in a randomized controlled trial receiving daily LNS, multiple micronutrients (MMN - multi vitamin and minerals combined supplement) or iron and folic acid supplements (IFA – 60 mg of iron and 400 μg of folic acid). The primary aim was to determine whether LNS supplementation improved antibody responses to malaria in pregnancy compared to IFA or MMN.

## Methods

### Study participants

From February 2011, 1391 pregnant women attending four antenatal clinics in Mangochi District, Malawi were recruited to a single-blinded randomized controlled trial of nutrient supplementation to improve pregnancy outcomes and child development (registration ID: NCT01239693 [23]). Mangochi District experiences holoendemic malaria transmission and a high prevalence of stunting (low height-for-age Z-score) and low weight-for-age among infants. Women <20 gestation weeks (gw) pregnant by ultrasound dating, aged >15 years of age and without any chronic health conditions were enrolled in the study following informed consent. They were randomly assigned to receive one tablet of IFA, one tablet of MMN or 20 g of LNS (containing 20 mg iron and 400 μg of folic acid) daily [23]. The final maternal visit of the trial was completed in February 2013. All participants received two doses of sulphadoxine-pyrimethamine (SP) malaria intermittent preventative treatment at enrolment and at 28–34 gestation weeks [23]. The follow up of the children of the trial is currently ongoing with the last visit expected to complete by December 2015; for further details [23,24].

### Ethics approval

The trial was approved by the College of Medicine Research and Ethics Committee of Malawi, and by Tampere University Hospital Ethics Committee, Finland. Laboratory studies were approved by the Melbourne Health Human Research Ethics Committee.

### Plasma sample collection and malaria detection

Blood plasma samples were collected from participants at study entry and at 36 gw. Malaria parasitaemia was diagnosed by light microscopy slide positivity, PCR or rapid diagnostic test (RDT) for asexual *P. falciparum* and/or mixed *Plasmodium* infections at enrolment and at 36 gw (PCR and RDT only). Parasitized red blood cells (pRBCs) were counted microscopically against 200 leucocytes under 100 X magnification. PCR was performed as previously described [25]. Briefly, genomic DNA was extracted from dried blood spots using Chelex®100 (Bio-Rad, CA, USA) and a quantitative real-time PCR assay was performed to amplify the *P. falciparum* lactate dehydrogenase (*pfldh*) gene. The samples were assayed in duplicate using a Bio-Rad CFX384 Touch Real-Time PCR detection system. Concentration of PfLDH was estimated using standard curves computed from amplification curves of a series of 10 g DNA extracts of parasite line 3D7 that were included in each assay. RDT was performed using Clearview® Malaria Combo (British Biocell International Ltd., Dundee, UK) which detects the proteins PfLDH and histidine-rich protein 2. The prevalence of malaria in the current cohort was 23.2% by RDT at baseline [23].

### Preparation of recombinant malaria antigens and schizont extract

Recombinant merozoite surface protein-1, MSP-1 19 kD (3D7 clone) and full-length MSP-2 (FC27 clone) antigens [26] were kindly provided by Paul Gilson (Burnet Institute, VIC, Australia) and Robin Anders (La Trobe University, Australia) respectively. In addition region III-V of erythrocyte binding antigen 175 (EBA 175) [27], *P. falciparum* reticulocyte binding homologue 2 (PfRh2) (construct PfRh2-2030) [28] and the full length MSP-3 recombinant proteins were prepared following established protocols. The schizont extract was prepared following the protocol described in [29].

### Measuring IgG and IgG subclass antibodies to merozoite and schizont antigens, and quantitating total plasma IgG

In brief, 0.5-2 μg/ml of each recombinant antigen was coated on to 384 well high protein binding black Optiplates (PerkinElmer Inc., Massachusetts, USA). The plates were washed with PBS/Tween 20 and non-specific binding blocked with 0.1% casein on the following day. The plates were then incubated with participant sera diluted at 1:1,000 in 0.1% casein and assayed in triplicates. Alexa Fluor 488-conjugated goat anti-human IgG (Life technologies, Australia) was used at 1:2,000 dilution to detect antigen-bound IgG. The fluorescence intensity (FI) of each well, which is proportional to the amount of IgG bound, was measured using BMG POLARstar Omega fluorimeter (BMG Labtech, Germany) in the 485–1 excitation and 520 emission spectra range.

Plasmas collected at enrolment and at 36 gw from 150 participants (50 from each supplementation group) were chosen by selecting alternate pairs of samples from 4 boxes containing equal numbers of samples from the three supplementation arms, blind to participant clinical data. These samples were used to measure IgG subclass antibodies to the above antigens. Antigens were coated at the same concentration on 384 well NUNC maxisorp plates (Thermo Fisher Scientific, VIC, Australia) and were incubated in triplicates with plasma on the following day at 1:250 dilution for IgG1and IgG3, and 1:50 for IgG2 and IgG4. Following incubation the plates were washed and incubated with respective mouse anti-human secondary antibodies (IgG1 clone HP6069, IgG2 clone HP6002, IgG3 HP6047 [Life Technologies, Australia] and IgG4 clone HP6023 (Merck Millipore, Darmstadt, Germany) at 1:1,000 dilution. Tertiary antibody horseradish peroxidase-conjugated goat anti-mouse IgG was added at 1:2,500 dilution followed by development of the assay using ABTS (2,2′-Azino-bis(3-ethylbenzothiazoline-6-sulfonic acid); SIGMA-Aldrich). The reaction was stopped using 1% sodium dodecyl sulphate, and absorbance was read on the BMG POLARstar Omega fluorimeter at 410 nm wavelength.

The total plasma IgG in the samples of the above 150 pregnant women were measured using the fluorescence assay method described above. Gamma-chain specific anti-human IgG capture antibody (SIGMA-Aldrich) was coated on to the plates at 0.5 μg/ml concentration and incubated with participants’ plasma diluted at 1:50,000. The concentration of IgG in each sample was determined using a purified human IgG isotype control (Life technologies) standard curve (1 μg/ml – 0.016 μg/ml).

Serial samples were run on the same assay. The mean FI (MFI) or optical density (OD) were reported as a percentage of the positive control standard curve following adjustment for inter and intra plate variability. The positive control was a pool of plasma collected from malaria-immune individuals. The positivity for IgG was determined as greater than mean plus three standard deviations of the negative control (60 plasma samples collected from Melbourne adults).

### Parasites and cell culture

Placental-binding *P. falciparum* isolate, CS2 which binds to chondroitin sulphate A (CSA) expressed in placenta tissue with upregulated expression of *var2csa* [30] and non-placental binding *P. falciparum* isolate, E8B which binds to ICAM-1 and CD36 [31] were cultured as previously described [32]. Pro-monocytic THP-1 cells were obtained from ATCC (Manassas, VA, USA; catalogue number TIB-202™) and maintained as previously described [33].

### Opsonic phagocytosis assay for measuring functional antibodies to placental-binding and non-placental-binding malaria variant surface antigens

In order to measure antibodies that opsonize pRBCs for phagocytic clearance a high throughput assay previously established in the current laboratory was used [33], with minor modifications [34], as described [32]. The percentage phagocytosis was determined relative to the positive control (pRBCs incubated with a pool of sera from patients with high levels of IgG to VSA). A participant was considered seropositive for opsonizing antibodies if the percentage phagocytosis of the samples was greater than the sum of the average and three standard deviations of the percentage phagocytosis of the negative controls.

### Measuring IgG to variant surface antigens expressed by placental-binding parasite isolate

Using published methods [32,35] IgG to VSA expressed by the placental-binding isolate was measured. Alexa Fluor® 647-conjugated donkey anti-rabbit antibody (Life Technologies) was used as the tertiary antibody at 4 μg/ml concentration in contrast to the previous methods. The geometric MFI of the samples were measured and adjusted for intra and inter-plate variability. The adjusted MFI for all samples were reported as a percentage of the positive control. Seropositivity to placental-binding VSA was calculated as geometric MFI greater than the mean and three standard deviations of the negative control.

### Statistical analyses

All statistical analyses were performed in Stata version 13.0 (SataCorp LP, Texas, USA). Figures were created using GraphPad Prism 5. Statistical analyses were performed according to the pre-planned analyses for the current study approved and published in [24]. Antibody levels were compared between supplementation groups LNS versus IFA and LNS versus MMN using Mann Whitney U test. Differences between antibody levels at the two time points were determined using Wilcoxon matched-pair test. The rate of change in antibody levels was calculated as difference between antibody levels at 36 gw and antibody levels at enrolment divided by the number of weeks of the study. A rate of change of 0, suggests no change in antibody levels per week of gestation. The rate of change in antibody levels was compared between LNS and IFA, and LNS and MMN, where IFA and MMN were the respective reference groups, by performing linear regression analysis.

Levels of antibody of each IgG subclass to merozoite and schizont antigens were compared between supplementation groups LNS versus IFA and LNS versus MMN using Mann Whitney U test. Comparisons were also made between antibody levels at enrolment and at 36 weeks using Wilcoxon matched-pair test.

The covariates of the association between supplementation type and malaria antibody immunity were determined based on their biological relevance. Covariates chosen were gravidity, maternal age, HIV infection, malaria infection, residence, socioeconomic status (SES), body mass index (BMI) and antibody levels at enrolment. Gravidity, maternal age, HIV and malaria infection were previously shown to associate with malaria antibody immunity [36,37]. Participants’ SES was calculated based on a scoring system for household assets (HHA) adapted from [38]. Participants whose HHA adjusted z-score was below the median were considered to have low SES. Body mass index (BMI) at enrolment was categorized into three groups as low (<18.5 kg/m^2^); normal (18.5-25 kg/m^2^) and increased (>25 kg/m^2^). All the variables were treated as categorical except for antibody levels at enrolment which was treated as a continuous variable. Likelihood ratio test was used to test whether the association between supplementation type and antibody levels at 36 weeks varied with covariates chosen for the study.

Nevertheless, all of the above variables (gravidity, maternal age, HIV, malaria infection at enrolment, location of residence, SES, BMI and antibody levels at enrolment were included in the multivariate regression models. Multivariate linear regression analyses for continuous outcomes (antibody levels at 36 weeks and the rate of change in antibody levels) and multivariate logistic regression analyses for categorical outcomes (seropositivity to malaria antigens). Differences in antibody levels among women with different BMI and SES groups were determined following multivariate linear regression analysis, adjusting for covariates including supplementation group, as detailed in the table footnotes. Associations were considered statistically significant if the p value is <0.05.

## Results

### Participant characteristics

Overall 1,391 women were enrolled in the iLiNS-DYAD-M study. Malaria immunity was only measured in the 1,009 enrolment and 36 weeks paired plasma samples that were available. The baseline characteristics of the participants across the supplementation arms were comparable (Table 1). The mean (SD) gestational age in weeks at enrolment was 16.5 (2.1) for IFA, 16.5 (2.2) for MMN and 16.6 (2.2) for LNS groups (Table 1). Approximately 60.2% of women were multigravidae. Proportion of women with BMI <18.5 kg/m^2^ was 5.7% and MUAC < 23cm was 6.8%, who were identified as undernourished women in the current cohort. Thirteen percent of women were HIV infected. The proportion of participants lost to follow up between the supplementation groups was similar with an overall 6.04% lost to follow up [23].

**Table 1:** **Baseline characteristics of study participants**

**Characteristic**	**IFA (n = 325)**	**MMN (n = 347)**	**LNS (n = 337)**	**All**
Gestation weeks at enrolment, mean (SD)	16.5 (2.12)	16.5 (2.23)	16.6 (2.24)	16.5 (2.20)
Age, years	23 (20–28)	24 (20–29)	24 (20–28)	24 (20–28)
Gravidity				
-Primigravidae	64 (19.7%)	67 (19.2%)	68 (20.1%)	199 (19.8%)
-Secundigravidae	77 (23.7%)	64 (18.4%)	62 (18.3%)	202 (20.1%)
-Multigravidae	184 (56.6%)	214 (61.5%)	208 (61.5%)	606 (60.2%)
BMI, kg/m^2^;	21.5 (20.4-23.3)	21.6 (20.1-23.5)	21.7 (20.3- 23.7)	21.6 (20.3-23.5)
-BMI <18.5 kg/m^2^, n (%)	18 (5.6%)	18 (5.3%)	21 (6.3%)	57 (5.7%)
MUAC (cm),	26.0 (24.7-27.5)	25.7 (24.2- 27.7)	26.1 (24.6- 27.8)	26.0 (24.5-27.7)
-MUAC < 23cm, n (%)	23 (7.1%)	21 (6.1%)	24 (7.1%)	68 (6.8%)
Malaria parasitaemia				
- Microscopy	34 (10.5%)	36 (10.4%)	38 (11.3%)	108 (10.7%)
- PCR	81 (25.7%)	79 (23.4%)	86 (25.7%)	246 (24.9%)
- RDT	71 (21.8%)	83 (24.1%)	75 (22.5%)	229 (22.8%)
HIV infection, n (%)	47 (14.5%)	37 (10.7%)	46 (13.6%)	130 (13.0%)
Low Socioeconomic status, n (%)^a^	184 (56.7%)	187 (53.9%)	189 (56.1%)	560 (55.9%)

Data presented as median (Interquartile range), or number (%) unless stated.

^a^Socioeconomic status calculated based on a scoring system for house hold assets. Low socioeconomic status is defined as house hold Z-score < median −0.387.

*Abbreviations*: SD: standard deviation; RDT: rapid diagnostic test; BMI: body mass index; MUAC: mid-upper arm circumference, PCR: Polymerase chain reaction.

### Changes in antibody levels between enrolment and 36 weeks

**Figure 1:**
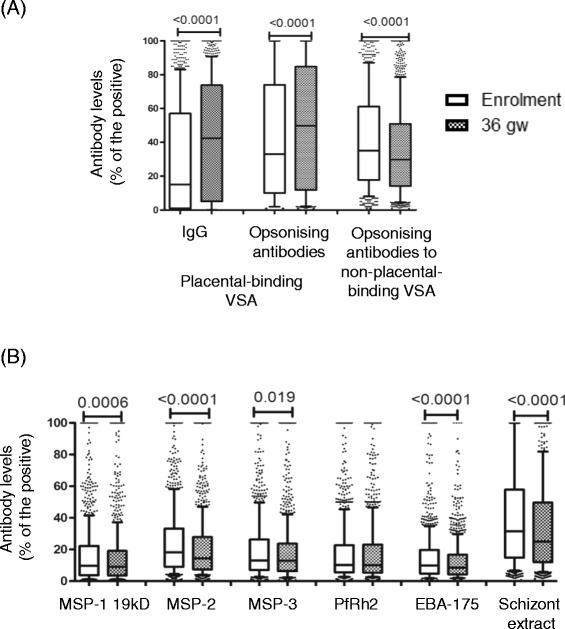
Antibodies to malaria antigens at enrolment and at 36 gestation weeks. **(A)** Antibodies to variant surface antigens expressed by placental-binding and non-placental-binding parasite isolates. **(B)** Antibodies to merozoite antigens and schizont extract. Data presented as box plots with the Y axis representing antibody levels as a percentage of the positive control. The whiskers denote the 10^th^ and the 90^th^ percentiles with outliers. Wilcoxon matched pairs test performed for antibody comparisons between enrolment and 36 gestation weeks. N = 1,009.

Opsonizing antibodies that promote phagocytic clearance and IgG against the VSA expressed by placental-binding isolate were significantly higher at 36 weeks compared to the levels at enrolment; median relative antibody levels, 49.7% compared to 32.7% at enrolment (p < 0.0001) and 42.3% compared to 15.0% at enrolment (p < 0.0001), respectively (Figure 1A). However, opsonizing antibodies to non-placental-binding isolate significantly declined, 29.8% at 36 weeks compared to 35.4% at enrolment (p < 0.0001) (Figure 1A). Antibody levels to MSP-1 19kD, MSP-2, MSP-3, EBA-175 and schizont extract were significantly reduced from enrolment to 36 weeks; median difference in relative antibody levels, 0.7% (p = 0.0006), 3.9% (p < 0.001), 0.2% (p = 0.019), 1.3% (p < 0.0001) and 6.5% (p < 0.0001), respectively (Figure 1B). The plasma IgG levels also significantly declined from enrolment to 36 weeks; median relative antibody levels, 6.9% to 4.6% (p < 0.0001) (See Additional file 1), similar to the decline in antibody responses to merozoite antigens and non-placental-binding parasite isolate VSA.

### Effect of nutrient supplementation on antibody immune responses to malaria

**Figure 2:**
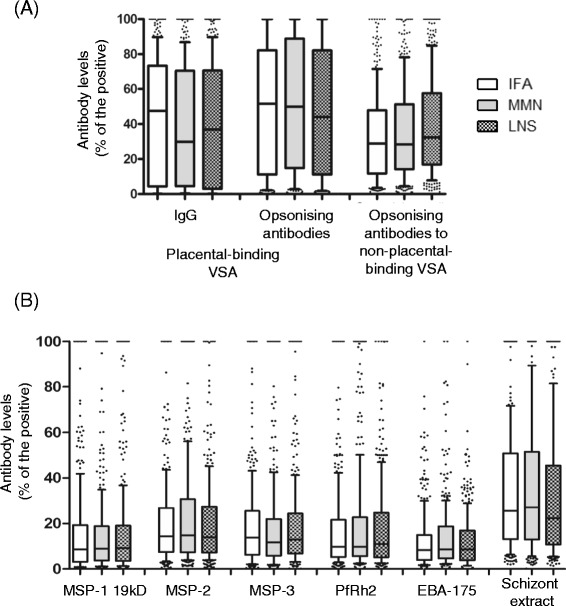
Antibodies to malaria antigens at 36 weeks categorized by supplementation type. **(A)** Antibodies to variant surface antigens expressed by placental-binding and non-placental-binding parasite isolates. **(B)** Antibodies to merozoite antigens and schizont extract. Data presented as box plots with the Y axis representing antibodies as a percentage of the positive control. The whiskers denote the 10^th^ and the 90^th^ percentiles with outliers. Mann Whitney U test performed for antibody comparisons between supplementation groups LNS versus IFA and LNS versus MMN. N = 1,009.

When categorized by the supplementation groups, no significant differences in the levels of antibody to placental-binding or non-placental-binding isolates were observed at 36 weeks (Figure 2A). There were also no significant differences in antibodies to merozoite or schizont antigens, when stratified by supplementation type (Figure 2B). Following adjustment for covariates, the levels of antibody to MSP-2 and PfRh2 at 36 weeks were significantly lower in the LNS group compared to MMN −3.98 (−7.59, −0.37) and −4.44 (−8.85, −0.03), respectively (See Additional file 2).

There were no differences in the prevalence of detectable antibody responses at 36 weeks by supplementation group (See Additional file 3). More than 85% of the pregnant women had antibodies to placental-binding VSA.

### Effect of supplementation on the rate of change in antibodies to malaria antigens

Significant differences in the rate of change in antibodies to parasite isolate VSA were not found between supplementation groups (Table 2), except for MSP-2. The rate of change in MSP-2 antibodies was significantly negatively associated with LNS group compared to MMN −0.25 (−0.46, −0.04) suggesting a faster decline in MSP-2 antibody responses in the LNS group (Table 2).

**Table 2:** **Association between nutrient supplementation and the rate of change in antibody levels**

	**Rate of change in antibody levels (Percentage antibody level change per gestation week, arbitrary units)**					
	**LNS (n = 337) compared to IFA (n = 325)**			**LNS (n = 337) compared to MMN (n = 347)**		
	**Unadjusted**	**Adjusted**	**Adjusted p-value**	**Unadjusted**	**Adjusted**	**Adjusted p-value**
IgG to placental-binding isolate VSA	−0.12 (−0.34, 0.11)	−0.08 (−0.38, 0.22)	0.599	0.13 (−0.09, 0.36)	0.26 (−0.04, 0.55)	0.089
Opsonizing antibodies to placental-binding solate VSA	−0.02 (−0.24, 0.19)	−0.11 (−0.41, 0.19)	0.469	0.06 (−0.14, 0.27)	0.02 (−0.26, 0.30)	0.864
Opsonizing antibodies to non-placental-binding isolate VSA	−0.002 (−0.12, 0.12)	0.01 (−0.16, 0.18)	0.888	−0.08 (−0.19, 0.03)	0.02 (−0.13, 0.18)	0.756
MSP-1 19kD	−0.02 (−0.17, 0.12)	0.006 (−0.18, 0.19)	0.953	−0.06 (−0.19, 0.08)	0.04 (−0.14, 0.21)	0.693
MSP-2	−0.12 (−0.27, 0.03)	−0.20 (−0.40, 0.02)	0.069	−0.15 (−0.32, 0.01)	−0.25 (−0.46, −0.04)*	0.021*
MSP-3	−0.18 (−0.36, −0.01)*	−0.21 (−0.44, 0.03)	0.080	−0.05 (−0.24, 0.14)	−0.01 (−0.28, 0.27)	0.970
PfRh2	0.07 (−0.13, 0.27)	0.10 (−0.17, 0.37)	0.461	−0.21 (−0.40, −0.02)*	−0.25 (−0.51, 0.02)	0.073
EBA-175	−0.06 (−0.18, 0.06)	−0.04 (−0.20, 0.13)	0.639	−0.07 (−0.20, 0.05)	−0.05 (−0.24, 0.14)	0.603
Schizont extract	−0.03 (−0.28, 0.23)	0.13 (−0.21, 0.48)	0.450	0.003 (−0.26, 0.27)	0.10 (−0.26, 0.47)	0.583

Results presented as regression coefficients (95% confidence interval) calculated by comparing the percentage rate of change in antibody levels in LNS group compared to IFA (reference group) and LNS group compared to MMN (reference group).

The percentage rate of change in antibody levels from enrolment to 36 gw were adjusted for gravidity, maternal age, HIV, malaria infection at enrolment, malaria infection at 36 weeks, bed net use, body mass index at enrolment, socioeconomic status and location of residence.

Statistical test: Multivariate linear regression analysis. Data reported as regression coefficient (95% confidence interval) and p-values. *Significant associations.

### Modification of the association between malaria antibody levels and supplementation group by covariates

Factors that influenced the relationship between antibody levels at 36 weeks and supplementation groups were investigated. This relationship was only affected by the antibody levels at enrolment (see Additional file 4). Levels at enrolment of opsonizing antibodies to non-placental binding and antibodies to MSP-2, MSP-3 and PfRh2 at enrolment significantly influenced the relationship between supplementation type and the levels of the respective antibodies at 36 weeks (0.001 < p < 0.006; Additional file 4). The effect modification by MSP-3 antibodies at enrolment was significant on the association between antibodies to MSP-3 at 36 weeks between LNS and IFA groups 3.44 (0.14, 6.75).

### Effect of nutrient supplementation on IgG subclass responses to malaria

For all the antigens tested, the highest subclass response was for IgG3 followed by IgG1 and IgG2 with almost undetectable IgG4 responses (See Additional file 5). When categorized by the supplementation groups, subclass responses were not significantly different. For many subclass responses, antibody levels declined significantly between enrolment and 36 weeks, in keeping with the decline in IgG to each antigen observed; this was most apparent for IgG3 antibodies.

### Influence of maternal nutrition status and socioeconomic status on malaria antibody responses

**Figure 3:**
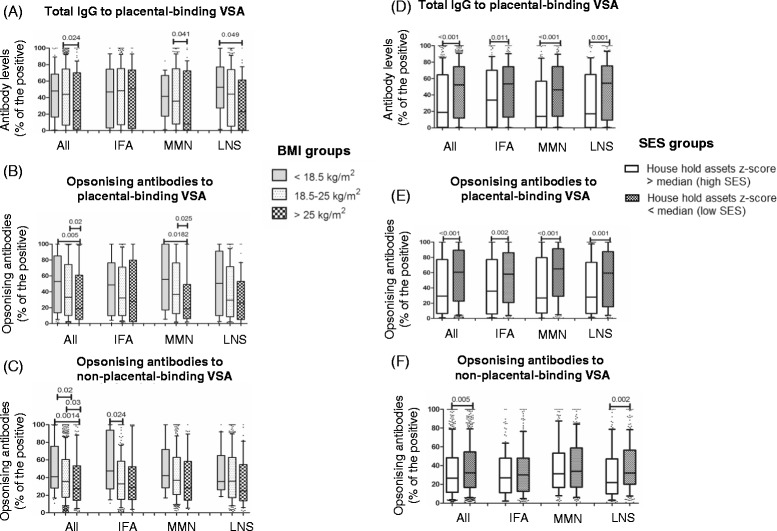
Antibodies at 36 weeks stratified by maternal body mass index and socioeconomic status. **(A-C)** Antibodies to variant surface antigens of placental-binding and non-placental-binding parasite isolates stratified by body mass index (BMI) groups and supplementation groups, **(D-F)** stratified by socioeconomic status (SES) and supplementation groups. BMI groups < 18.5 kg/m^2^, n = 57; 18.5-25 kg/m^2^, n = 822; >25 kg/m^2^, n = 122. High SES  = 441 and low SES n = 560.

Overall, higher levels of malaria antibodies were observed for women with low BMI compared to the other BMI groups (Figure 3A-C). This difference was significant for opsonic antibodies to both placental-binding; median (p-value), 52.8% (p = 0.005) and non-placental-binding isolates 41.0% (p = 0.001) (Figure 3B-C), and was not affected by the supplementation type. When antibody responses were stratified by supplementation groups, significant differences in antibody levels between BMI groups within the same supplementation group were observed, although these associations were not strongly consistent (Figure 3).

Following adjustment for covariates, opsonizing antibodies to the placental-binding isolate, antibodies to PfRh2 and schizont extract were significantly negatively associated with BMI, a unit (1 kg/m^2^) increase in BMI is associated with a decrease in relative antibody levels by −1.04% (95% CI: −1.84, −0.24), −0.66% (−1.32, −0.004) and −1.22% (−2.08, −0.37) respectively (Table 3).

**Table 3:** **Relationship of maternal nutritional status and socioeconomic status to malaria antibody immunity at 36 weeks**

	**Body mass index at enrolment (kg/m** ^**2**^ **)**			**Socioeconomic status (high/low)**		
	**Unadjusted**	**Adjusted** ^**a**^	**Adjusted p-value** ^**a**^	**Unadjusted**	**Adjusted** ^**b**^	**Adjusted p-value** ^**b**^
IgG to placental-binding isolate VSA	−0.88 (−1.66, −0.10)*	−0.72 (−1.57, 0.12)	0.095	−13.9 (−18.25, −9.58)*	−9.54 (−14.74, −4.35)*	<0.001*
Opsonizing antibodies to placental-binding isolate VSA	−0.86 (−1.65, −0.06)*	−1.04 (−1.84, −0.24)*	0.011*	−14.7 (−19.18, −10.27)*	−5.47 (−10.43, −0.50)*	0.031*
Opsonizing antibodies to non-placental-binding isolate VSA	−0.53 (−1.11, 0.06)	0.25 (−0.20, 0.70)	0.272	−4.07 (−7.42, −0 .70)*	−0.59 (−3.38, 2.20)	0.678
MSP-1 19kD	−0.08 (−0.53, 0.37)	0.45 (−0.02, 0.92)	0.060	−1.62 (−4.19, 0.96)	−1.39 (−4.36, 1.57)	0.355
MSP-2	−0.40 (−0.88, 0.08)	0.14 (−0.37, 0.65)	0.595	−2.41 (−5.12, 0.30)	−0.26 (−3.46, 2.94)	0.874
MSP-3	−0.01 (−0.47, 0.44)	0.06 (−0.57, 0.69)	0.856	−0.83 (−3.43, 1.77)	0.38 (−3.55, 4.31)	0.849
PfRh2	−0.30 (−0.77, 0.17)	−0.66 (−1.32, −0.004)*	0.051*	−0.49 (−3.17, 2.18)	0.49 (−3.64, 4.61)	0.817
EBA-175	−0.15 (−0.46, 0.16)	0.11 (−0.28, 0.50)	0.573	−1.12 (−2.89, 0.65)	−0.22 (−2.66, 2.22)	0.858
Schizont extract	−0.64 (−1.35, 0.07)	−1.22 ( −2.08, −0.37)*	0.005*	−5.17 (−9.24, −1.10)*	−0.55 (−6.12, 5.01)	0.845

Data presented as regression coefficient (95% confidence interval).

^a^Percentage antibody levels at 36 gestation week adjusted for gravidity, maternal age, HIV, malaria infection at enrolment, bed net use, location of residence, antibody levels at enrolment, socioeconomic status, supplementation group.

^b^Percentage antibody levels at 36 gestation week adjusted for gravidity, maternal age, HIV, malaria infection at enrolment, bed net use, location of residence, antibody levels at enrolment, body mass index at enrolment, supplementation group.

Statistical test: Multivariate linear regression analysis. Data reported as regression coefficient (95% confidence interval) and p-values. *Significant associations.

In a univariate analysis, women with a low SES had significantly higher antibody levels to VSA compared to women with high SES, including both opsonizing antibodies and IgG to placental-binding VSA (p < 0.001), and opsonizing antibodies to non-placental-binding VSA (p = 0.005) (Figure 3D-F). Both IgG and opsonizing antibodies to the placental-binding isolate (but not other responses) remained significantly associated with SES following adjustment for covariates, coefficients −9.54 (−14.74, −4.35) and −5.47 (−10.43, −0.50) respectively (Table 3).

## Discussion

The association between nutrient supplementation and malaria immunity was evaluated in 1,009 pregnant women enrolled in a randomized controlled trial comparing LNS, MMN and IFA. Ingestion of LNS was not associated with significant increases in malaria antibody levels, a faster rate of change of antibody levels from enrolment to 36 weeks, or likelihood of seropositivity to malaria antigens. This study is the first to investigate the relationship between nutrient supplementation and malaria immunity in detail.

Antibodies were measured against VSA expressed by a placental-binding and non-placental-binding parasite isolate, merozoite antigens and schizont extract. Antibodies to placental-binding VSA reduced the risk of placental malaria by inhibiting cytoadhesion to receptors [37], and by promoting their phagocytic clearance [39]. Antibodies to merozoite antigens can prevent invasion of pRBCs [29] inhibit intra-erythrocytic development and opsonize merozoites for phagocytic clearance [40,41] contributing to the protection against clinical malaria.

A strong increase in both total and opsonizing antibodies to placental-binding VSA from study entry to 36 weeks was observed while antibodies to merozoite anti6gens and non-placental-binding isolates declined. Several explanations are possible. First, most parasite isolates infecting pregnant women express antigens that are unique and are exclusively expressed during pregnancy [42]. Antibodies induced against such antigens may have a longer half-life than those directed against merozoite antigens [43]. Second, reduction in total plasma IgG at 36 weeks suggests haemodilution may have an impact on antibody levels to non-placental-binding malaria antigens; however, antibodies to tetanus and measles show very little decline during pregnancy [43] suggesting haemodilution is not always a significant factor. Third, antibodies to antigens expressed by a placental-binding isolate appear to be maintained from previous pregnancies, and the majority of women in the cohort were multigravidae with high levels of these antibodies at study entry; exposure to new infections might rapidly boost antibody responses. By contrast, antibodies to merozoite antigens have been reported to fluctuate markedly with exposure during pregnancy [43]. Of note, all participants received repeated doses of SP; parasite resistance to this agent in Malawi is common, but not high-grade [44]. Parasites with SP resistance demonstrate mutations in the genes encoding *P. falciparum* dihydrofolate reductase (DHFR) and dihydropteroate (DHPS). In Malawian pregnant women quintuple mutations of DHFR and DHPS are highly prevalent, however the prevalence of more severe sextuple mutant DHPS 581 which is associated with increased placental inflammation is uncommon [45].

The type of supplementation had no strong or consistent influence on malaria antibody levels, however a regression analysis showed a decline in antibody responses to MSP-2 and PfRh2 from enrolment to 36 weeks, and an increased rate of reduction in antibody levels to MSP-2, in women who received LNS compared to MMN. Given the large number of comparisons reported, these differences in the adjusted models could be due to chance. Similar differences in other anti-merozoite antibodies were not observed, and the significance of the changes is unknown. The current study also reported the effects of nutrient supplementation on antibody responses in subsets of women at high risk of undernutrition and infection; those with poor nutritional status and low SES. A negative relationship was observed between malaria antibody levels and maternal BMI and SES. Overall, women with a low BMI at study entry had higher antibody levels to VSA than did women with normal or increased BMI. Supplementation type did not influence the relationship between antibody responses and BMI. Of all the antibody types being tested the opsonizing antibodies to VSA were the most strongly related to BMI and SES in the current cohort, suggesting that these women might be exposed to more malaria (Nkhoma M *et al.*, unpublished work) during the current pregnancy, thereby eliciting pregnancy-specific immunity. Poorer women could have more exposure to malaria due to poor housing that allows entry of mosquitoes, and they may be less likely to own insecticide-treated bed nets. In addition poverty could lead to poor nutrient intake due to inability to afford food especially during the hunger season.

The study had several limitations. The timing of nutritional interventions may be critical; supplementation starting prior to conception and continuing through out pregnancy and lactation may offer more benefits. In the current study the intervention started on average at 16.5 gw, following malaria peak prevalence, at around 12-17gw. Therefore the maximal benefits of the supplementation on malaria antibody acquisition may have been missed. Primigravidae constituted only 20% of participants, limiting our ability to examine acquisition of pregnancy-specific immunity in this group. Samples were missing for 382 of 1,391 women enrolled. Potential causes include failure to attend the 36-week visit, or pre-term deliveries (prior to 37 weeks). In addition unavailability of participant data for some of the covariates may have reduced the power of the multiple regression analyses. For analysis of BMI and malaria immunity, stratification of participants reduced the study power: for instance only 57 pregnant women were in the low BMI group.

## Conclusions

Overall the current study concluded that nutrient supplementation of pregnant women was not associated with broad changes in antibody responses to malaria. This study is the first to investigate interactions between nutrition and malaria immunity in pregnancy and further studies must be conducted to determine the role of nutrient supplementation on other components of host immune system such as cell-mediated immunity, to determine the overall influence of nutrient supplementation on malaria susceptibility and malaria immunity.

## Additional files

Additional file 1:
**Plasma IgG at enrolment and at 36 gestation weeks.** Data presented as box plot with the whiskers representing 10^th^ and the 90^th^ percentiles with outliers. Y axis presents plasma IgG concentration in g/L calculated from the standard curve created by purified human IgG. Wilcoxon matched pair test performed for comparison of antibody levels between enrolment and 36 weeks. Total plasma IgG significantly reduced (p < 0.0001) by 36 weeks compared to the levels at enrolment. Plasma IgG concentration was measured in a subset of women (n = 150). Additional file 2:
**Association between nutrient supplementation and antibodies to malaria at 36 gestation weeks.**
Additional file 3:
**Seropositivity to malaria antigens at 36 gestation weeks.**
Additional file 4:
**Antibody levels at enrolment as effect modifiers of the relationship between antibody levels at 36 weeks and supplementation group.**
Additional file 5:
**IgG subclass measurements for merozoite antigens and schizont extract at enrolment and at 36 weeks.** Antibody IgG subclasses presented as optical density (OD) measured at 410nm, categorized by supplementation groups for antigens MSP-1 19kD, MSP-2, MSP-3, PfRh2, EBA-175 and schizont extract (A-F). Kruskal Wallis test performed to determine subclass level differences between supplementation groups (IFA and LNS, MMN and LNS). Wilcoxon matched-pair test was performed for comparing subclass levels between enrolment and 36 gw. No significant differences in subclass levels across the supplementation groups were observed. Subclass levels at enrolment and 36 weeks were significantly different, denoted as P < 0.05 *, p < 0.005 **, p < 0.0001 ***. IFA (n = 50), MMN (n = 50), LNS (n = 50).

## Abbreviations

VSA: variant surface antigens
LNS: lipid-based nutrient supplement
gw: gestation weeks
MMN: multiple micronutrient supplement
IFA: iron and folic acid
HHA: household assets
SES: socioeconomic status
BMI: body mass index
MUAC: mid-upper arm circumference
MSP: merozoite surface protein
EBA: erythrocyte binding antigen
PfRh: *Plasmodium falciparum* reticulocyte binding homologue
