# Supplementary material for: The impact of lipid-based nutrient supplementation on anti-malarial antibodies in pregnant women in a randomized controlled trial
Source: Malar J. 2015 May 10;14:193. doi: 10.1186/s12936-015-0707-2 (PMC4438573; doi:10.1186/s12936-015-0707-2)
Supplement: Additional file 1: — Plasma IgG at enrolment and at 36 gestation weeks. Data presented as box plot with the whiskers representing 10th and the 90th percentiles with outliers. Y axis presents plasma IgG concentration in g/L calculated from the standard curve created by purified human IgG. Wilcoxon matched pair test performed for comparison of antibody levels between enrolment and 36 weeks. Total plasma IgG significantly reduced (p < 0.0001) by 36 weeks compared to the levels at enrolment. Plasma IgG concentration was measured in a subset of women (n = 150). [file 12936_2015_707_MOESM1_ESM.pdf]

Additional file 1: Plasma IgG at enrolment and at 36 gestation weeks

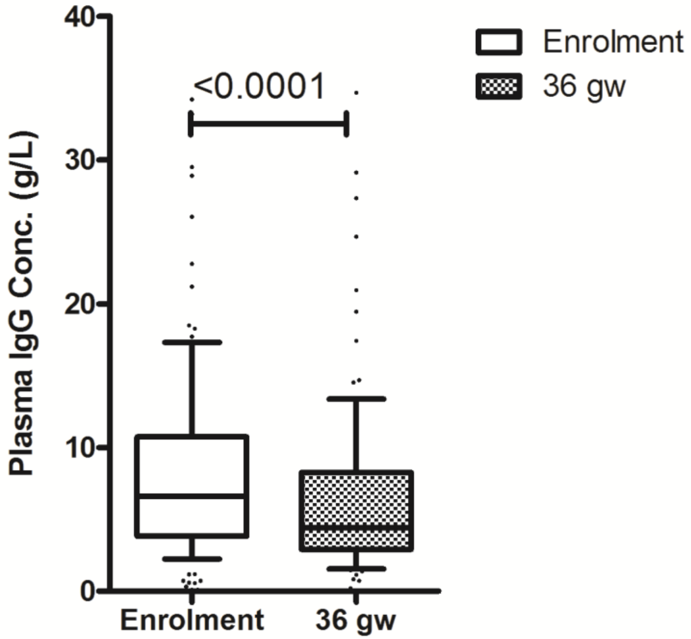

Data presented as box plot with the whiskers representing 10th and the 90th percentiles with outliers. Y axis presents plasma IgG concentration in g/L calculated from the standard curve created by purified human IgG. Wilcoxon matched pair test performed for comparison of antibody levels between enrolment and 36 weeks. Total plasma IgG significantly reduced ($p<0.0001$) by 36 weeks compared to the levels at enrolment. Plasma IgG concentration was measured in a subset of women (n=150).
