# Supplementary material for: The impact of lipid-based nutrient supplementation on anti-malarial antibodies in pregnant women in a randomized controlled trial
Source: Malar J. 2015 May 10;14:193. doi: 10.1186/s12936-015-0707-2 (PMC4438573; doi:10.1186/s12936-015-0707-2)
Supplement: Additional file 2: — Association between nutrient supplementation and antibodies to malaria at 36 gestation weeks. [file 12936_2015_707_MOESM2_ESM.docx]

**Additional file 2:** Association between nutrient supplementation on antibodies to malaria at 36 gestation weeks

|  | **Antibody levels at 36 weeks** | | | | | |
| --- | --- | --- | --- | --- | --- | --- |
|  | **LNS (n=337) compared to IFA (n=325)** | | | **LNS (n=337) compared to MMN (n=347)** | | |
| **Outcome** | **Unadjusted** | **Adjusted** | **Adjusted p-value** | **Unadjusted** | **Adjusted** | **Adjusted p-value** |
| IgG to placental-binding isolate VSA | -3.05 (-8.46, 2.35) | -2.22 (-7.80, 3.36) | 0.434 | 0.21 (-5.11, 5.54) | 4.78 (-0.63, 10.18) | 0.083 |
| Opsonising antibodies to placental-binding isolate VSA | -2.18 (-7.72, 3.36) | -3.65 (-9.07, 1.77) | 0.186 | -3.97 (-9.46, 1.52) | -1.24 (-6.19, 3.72) | 0.624 |
| Opsonising antibodies to non-placental-binding isolate VSA | -3.10 (-7.10, 1.00) | 0.06 (-3.00, 3.12) | 0.971 | -3.10 (-7.10, 1.00) | -0.17 (-2.98, 2.64) | 0.906 |
| MSP-1 19kD | -0.77 (-3.86, 2.31) | 0.09 (-3.17, 3.56) | 0.955 | -1.13 (-4.17, 1.91) | 0.86 (-2.26, 3.98) | 0.589 |
| MSP-2 | 0.12 (-2.95, 3.18) | -1.75 (-5.08, 1.58) | 0.303 | -3.03 (-6.37, 0.32) | -3.98 (-7.59, -0.37) | 0.031* |
| MSP-3 | 0.02 (-3.10, 3.15) | -1.75 (-5.82, 2.31) | 0.397 | 0.49 (-2.67, 3.64) | -0.85 (-3.34, 5.04) | 0.691 |
| PfRh2 | 1.38 (-1.63, 4.39) | 0.06 (-3.81, 3.93) | 0.975 | -1.26 (-4.59, 2.07) | -4.44 (-8.85, -0.03) | 0.049* |
| EBA-175 | -0.12 (-2.17, 1.94) | -0.50 (-2.87, 1.86) | 0.675 | -1.24 (-3.37, 0.90) | -1.26 (-3.93, 1.41) | 0.353 |
| Schizont extract | -1.64 (-6.46, 3.18) | 1.05 (-4.72, 6.81) | 0.721 | -3.94 (-8.91, 1.03) | -0.14 (-6.19, 5.92) | 0.965 |

Data reported as regression coefficient (95% confidence interval) and p-values reported. *Significant associations Antibody levels at 36 gestation week adjusted for gravidity, maternal age, HIV, malaria infection at enrolment, bed net use, socioeconomic status, location of residence and antibody levels at enrolment
