# Supplementary material for: The impact of lipid-based nutrient supplementation on anti-malarial antibodies in pregnant women in a randomized controlled trial
Source: Malar J. 2015 May 10;14:193. doi: 10.1186/s12936-015-0707-2 (PMC4438573; doi:10.1186/s12936-015-0707-2)
Supplement: Additional file 3: — Seropositivity to malaria antigens at 36 gestation weeks. [file 12936_2015_707_MOESM3_ESM.doc]

**Additional file 3:** Seropositivity to malaria antigens at 36 gestation weeks

|  | **Proportion of seropositives by supplementation group^a^** | | | | **LNS (n=337) compared to IFA (n=325)^c^** | | | **LNS (n=337) compared to MMN (n=347)^c^** | | |
| --- | --- | --- | --- | --- | --- | --- | --- | --- | --- | --- |
|  | **IFA** | **MMN** | **LNS** | **P-value^b^** | **OR (95 % CI)** | **Adjusted OR (95 % CI)** | **Adjusted P-value^d^** | **OR (95 % CI)** | **Adjusted OR (95 % CI)** | **Adjusted P-value^d^** |
| IgG to placental-binding isolate VSA | 273/312 (87.5%) | 307/334 (91.9%) | 286/321 (89.1%) | 0.176 | 1.08 (0.85, 1.38) | 1.51 (0.90, 2.52) | 0.119 | 0.72 (0.42, 1.22) | 0.94 (0.34, 2.58) | 0.907 |
| Opsonising antibodies to placental-binding isolate VSA | 307/325 (94.4%) | 327/347 (94.2%) | 313/336 (93.1%) | 0.751 | 0.89 (0.65, 1.23) | 1.20 (0.73, 2.00) | 0.471 | 0.83 (0.45, 1.55) | 1.82 (0.67, 4.94) | 0.242 |
| Opsonising antibodies to non-placental-binding isolate VSA | 286/321 (89.1%) | 320/345 (92.8%) | 306/336 (91.1%) | 0.256 | 1.12 (0.86, 1.44) | 1.26 (0.79, 2.00) | 0.334 | 0.80 (0.46, 1.39) | 0.97 (0.38, 2.48) | 0.956 |
| MSP-1 19kD | 193/318 (60.7%) | 217/343 (63.3%) | 208/333 (62.5%) | 0.786 | 1.04 (0.89, 1.22) | 1.00 (0.77, 1.30) | 0.996 | 0.97 (0.71, 1.32) | 0.87 (0.51, 1.50) | 0.626 |
| MSP-2 | 220/318 (69.2%) | 248/343 (72.3%) | 229/333 (68.8%) | 0.548 | 0.99 (0.84, 1.17) | 0.87 (0.66, 1.15) | 0.334 | 0.84 (0.61, 1.17) | 0.58 (0.33, 1.01) | 0.054 |
| MSP-3 | 217/318 (68.2%) | 223/343 (65.0%) | 233/333 (70.0%) | 0.376 | 1.04 (0.88, 1.23) | 0.97 (0.75, 1.25) | 0.792 | 1.25 (0.91, 1.73) | 1.17 (0.72, 1.88) | 0.526 |
| EBA-175 | 130/318 (40.9%) | 159/343 (46.4%) | 139/333 (41.7%) | 0.305 | 1.02 (0.87, 1.19) | 0.96 (0.75, 1.22) | 0.724 | 0.83 (0.61, 1.12) | 0.77 (0.47, 1.26) | 0.302 |
| PfRh2 | 186/318 (58.5%) | 197/343 (57.4%) | 198/333 (59.5%) | 0.867 | 1.02 (0.87, 1.19) | 1.03 (0.82, 1.29) | 0.811 | 0.90 (0.57, 1.40) | 0.87 (0.55, 1.39) | 0.570 |
| Schizont extract | 213/249  (85.5%) | 236/266  (88.7%) | 215/256  (84.0%) | 0.279 | 0.94 (0.74, 1.20) | 1.06 (0.73, 1.53) | 0.754 | 0.67 (0.40, 1.11) | 0.77 (0.35, 1.72) | 0.529 |

^a^Data presented as number (proportion of women) seropositive to each malaria antigens at 36 gw. Seropositivity calculated as sample mean fluorescence intensity (FI) > Mean FI of negative control + 3X standard deviation of the negative controls. ^b^P-value calculated following Chi^2^ test between the proportion of participants with seropositivity to each antigen ^c^Data presented as odds ratios of seropositivity between LNS group compared to IFA (reference group) and LNS compared to MMN (reference group) (95% confidence interval) unadjusted and adjusted for confounders gravidity, maternal age, HIV, malaria infection at enrolment, bed net use, socioeconomic status, location of residence and antibody levels at enrolment ^d^P-value adjusted for the above confounders
