# Supplementary material for: The impact of lipid-based nutrient supplementation on anti-malarial antibodies in pregnant women in a randomized controlled trial
Source: Malar J. 2015 May 10;14:193. doi: 10.1186/s12936-015-0707-2 (PMC4438573; doi:10.1186/s12936-015-0707-2)
Supplement: Additional file 4: — Antibody levels at enrolment as effect modifiers of the relationship between antibody levels at 36 weeks and supplementation group. [file 12936_2015_707_MOESM4_ESM.doc]

**Additional file 4:** Antibody levels at enrolment as effect modifiers of the relationship between antibody levels at 36 weeks and supplementation group

|  |  | **Comparison between LNS and IFA group** | | **Comparison between LNS and MMN group** | |
| --- | --- | --- | --- | --- | --- |
| **Effect modification of antibody levels at 36 weeks by** | **Interaction test p-value^a^** | **Differences in mean (95 % CI)** | **P-value^b^** | **Difference in mean (95 % CI)** | **P-value^b^** |
| Opsonising antibodies to non-placental-binding isolate VSA | 0.004* | 2.08 (-2.28, 6.45) | 0.348 | -1.50 (-5.77, 2.77) | 0.491 |
| MSP-2 at enrolment | 0.006* | 2.38 (-1.15, 5.91) | 0.186 | -0.17 (-3.82, 3.49) | 0.929 |
| MSP-3 at enrolment | 0.005* | 3.44 (0.14, 6.75) | 0.041* | 1.38 (-2.04, 4.80) | 0.428 |
| Rh2A9 at enrolment | 0.001* | 0.05 (-3.24, 3.35) | 0.974 | 2.67 (-0.30, 5.65) | 0.078 |

Data presented only for interactions which were significantly affecting the relationship between ^a^P-value calculated using likelihood ratio test; results for antibody measures at enrolment giving non-significant interactions with antibody levels at 36 weeks are not shown. ^b^P-value calculated using ANOVA comparing mean differences between supplemenation groups.
