# Supplementary material for: The impact of lipid-based nutrient supplementation on anti-malarial antibodies in pregnant women in a randomized controlled trial
Source: Malar J. 2015 May 10;14:193. doi: 10.1186/s12936-015-0707-2 (PMC4438573; doi:10.1186/s12936-015-0707-2)
Supplement: Additional file 5: — IgG subclass measurements for merozoite antigens and schizont extract at enrolment and at 36 weeks. Antibody IgG subclasses presented as optical density (OD) measured at 410nm, categorized by supplementation groups for antigens MSP-1 19kD, MSP-2, MSP-3, PfRh2, EBA-175 and schizont extract (A-F). Kruskal Wallis test performed to determine subclass level differences between supplementation groups (IFA and LNS, MMN and LNS). Wilcoxon matched-pair test was performed for comparing subclass levels between enrolment and 36 gw. No significant differences in subclass levels across the supplementation groups were observed. Subclass levels at enrolment and 36 weeks were significantly different, denoted as P < 0.05 *, p < 0.005 **, p < 0.0001 ***. IFA (n = 50), MMN (n = 50), LNS (n = 50). [file 12936_2015_707_MOESM5_ESM.pdf]

Additional file 5: IgG subclass measurements for merozoite antigens and schizont extract at enrolment and at 36 weeks

(A) **MSP-1 19kD**

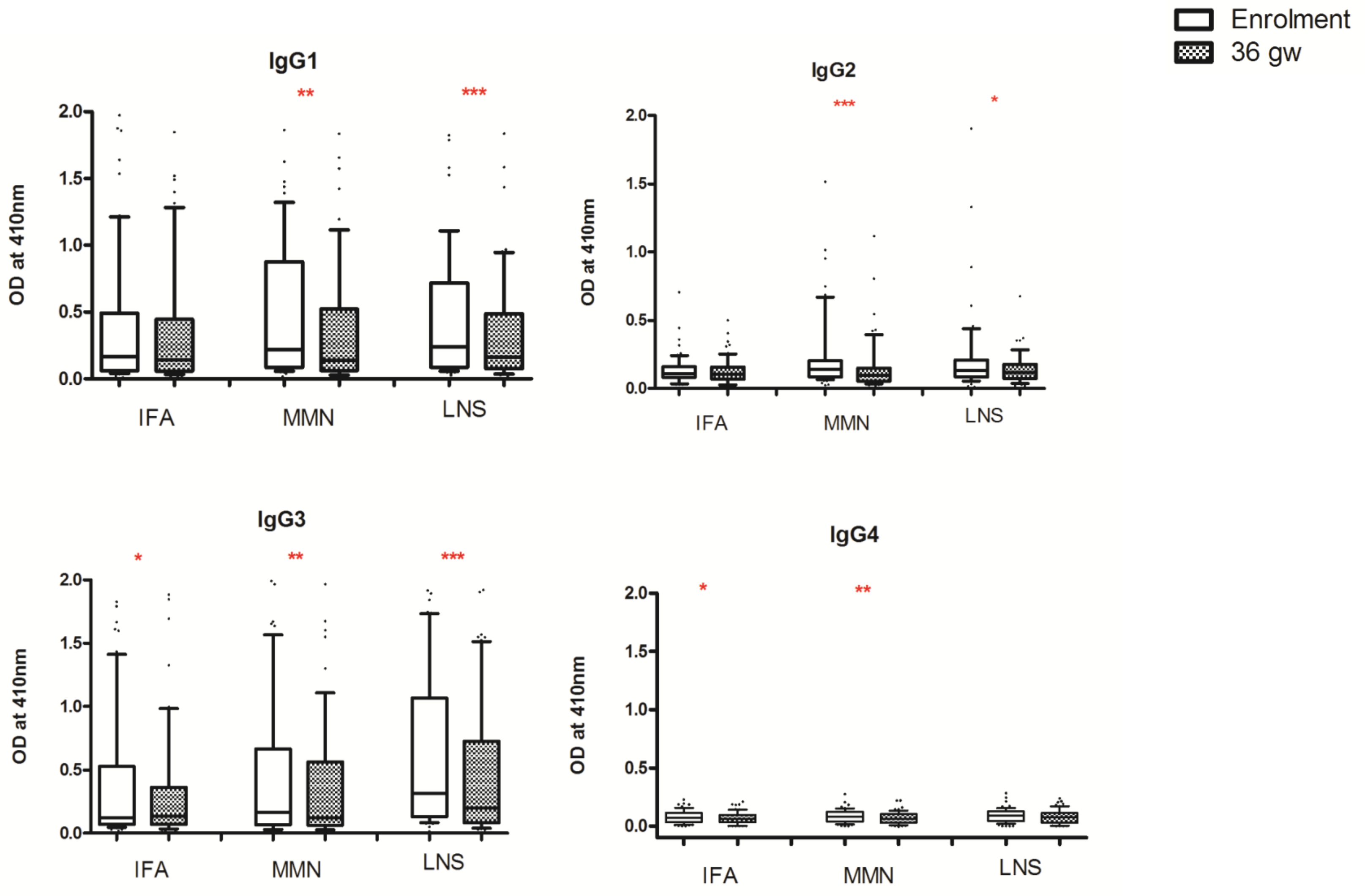

(B) **MSP-2**

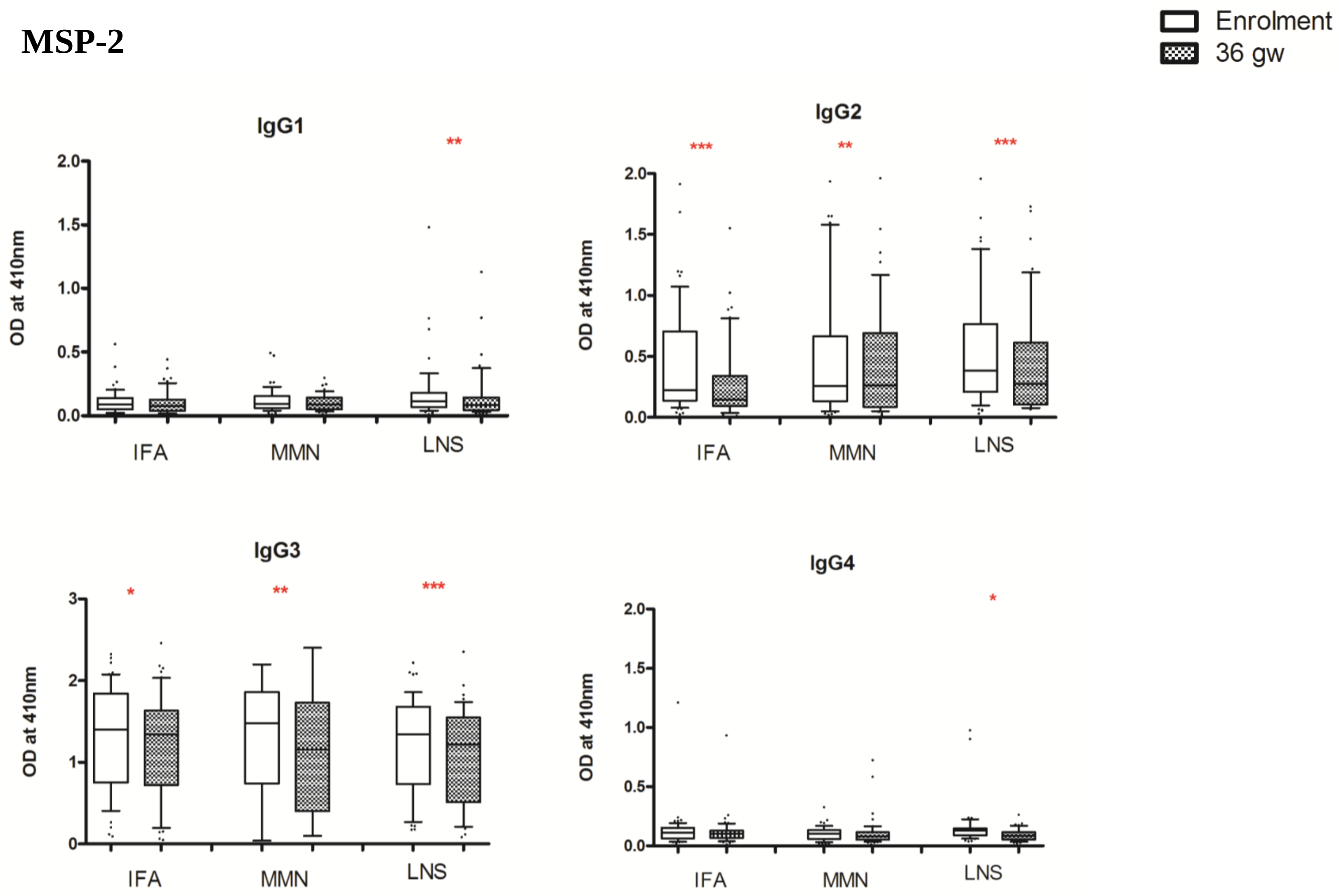

(C) **MSP-3**

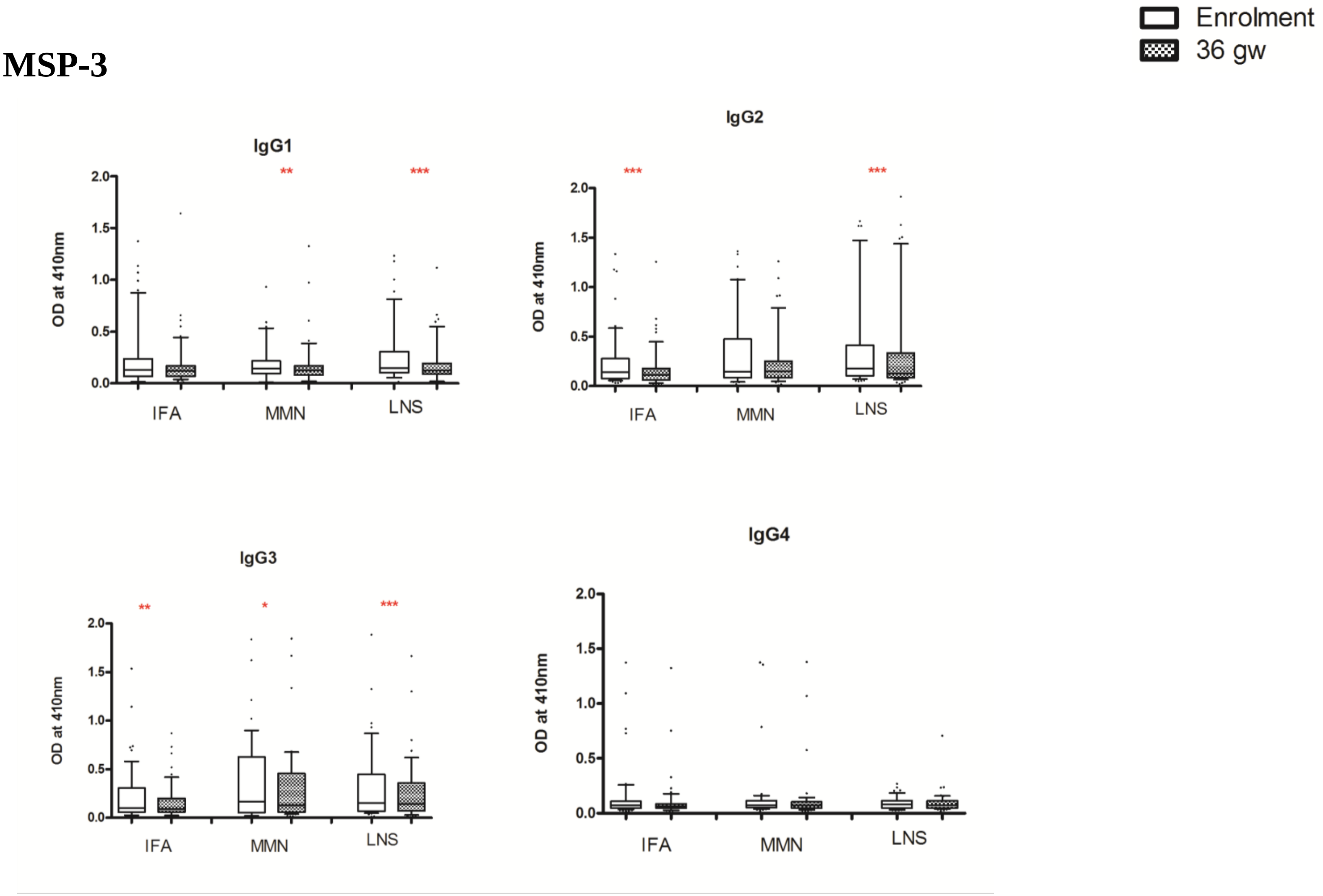
Enrolment
36 gw
IgG1
IgG2
IgG3
IgG4
OD at 410nm
2.0
1.5
1.0
0.5
0.0
**
***
*
IFA
MMN
LNS

(D) **PfRh2**

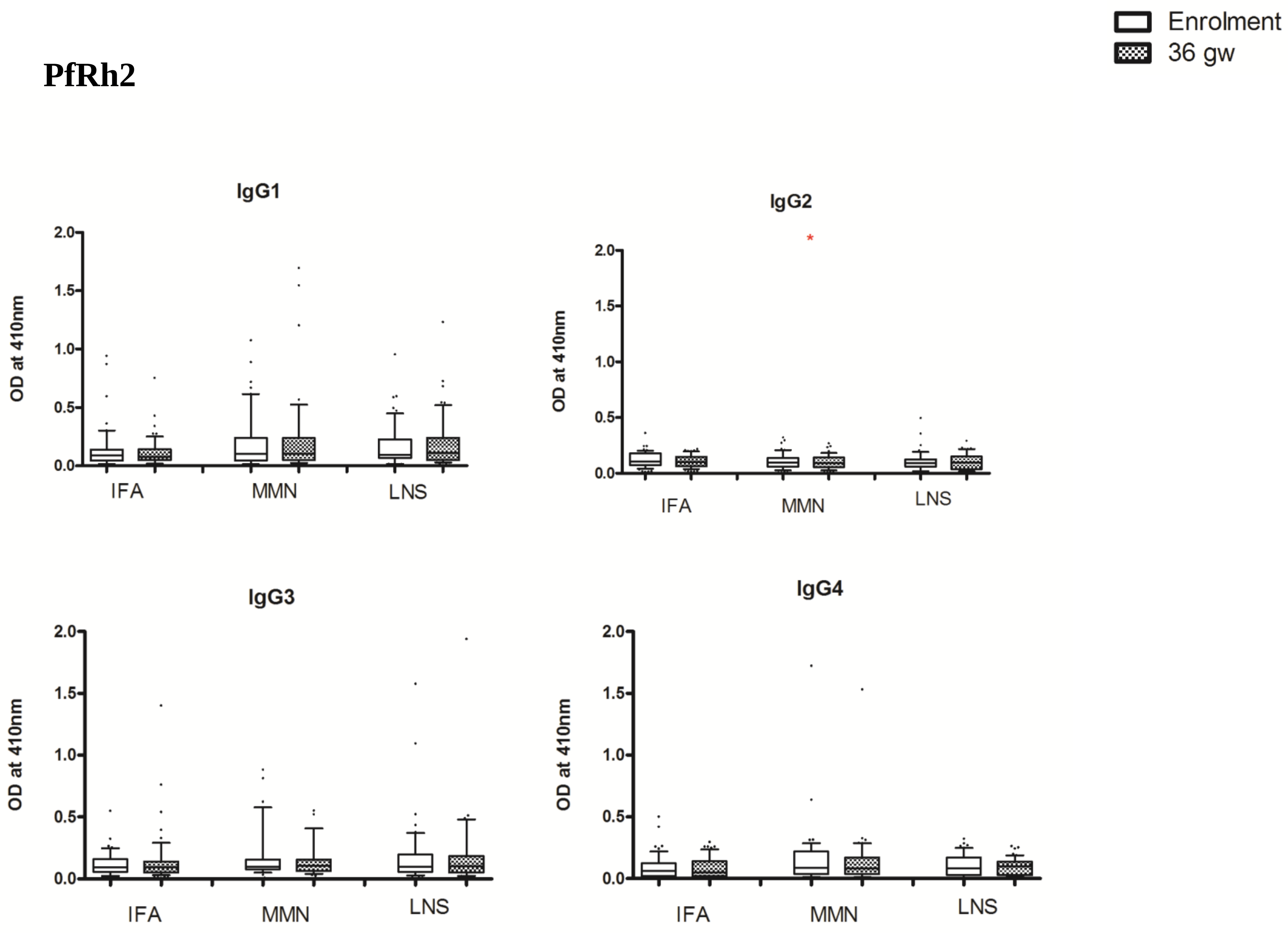

Enrolment
36 gw
IgG1
OD at 410nm
2.0
1.5
1.0
0.5
0.0
IFA
MMN
LNS
IgG2
*
IgG3
IgG4

(E) **EBA-175**

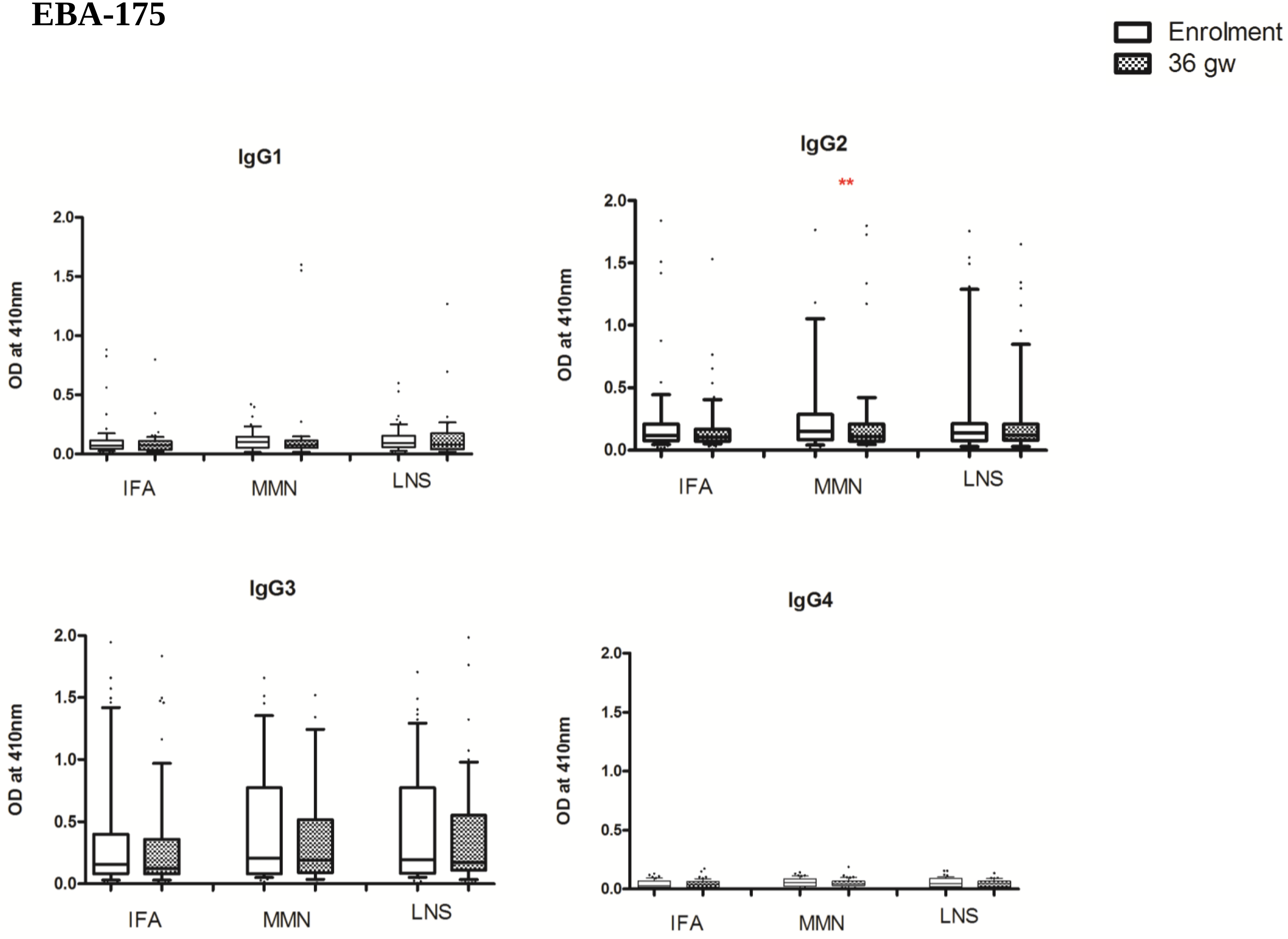
Enrolment
36 gw
IgG1
OD at 410nm
2.0
1.5
1.0
0.5
0.0
IFA
MMN
LNS
IgG2
**
OD at 410nm
2.0
1.5
1.0
0.5
0.0
IFA
MMN
LNS
IgG3
OD at 410nm
2.0
1.5
1.0
0.5
0.0
IFA
MMN
LNS
IgG4
OD at 410nm
2.0
1.5
1.0
0.5
0.0
IFA
MMN
LNS

(F) **Schizont extract**

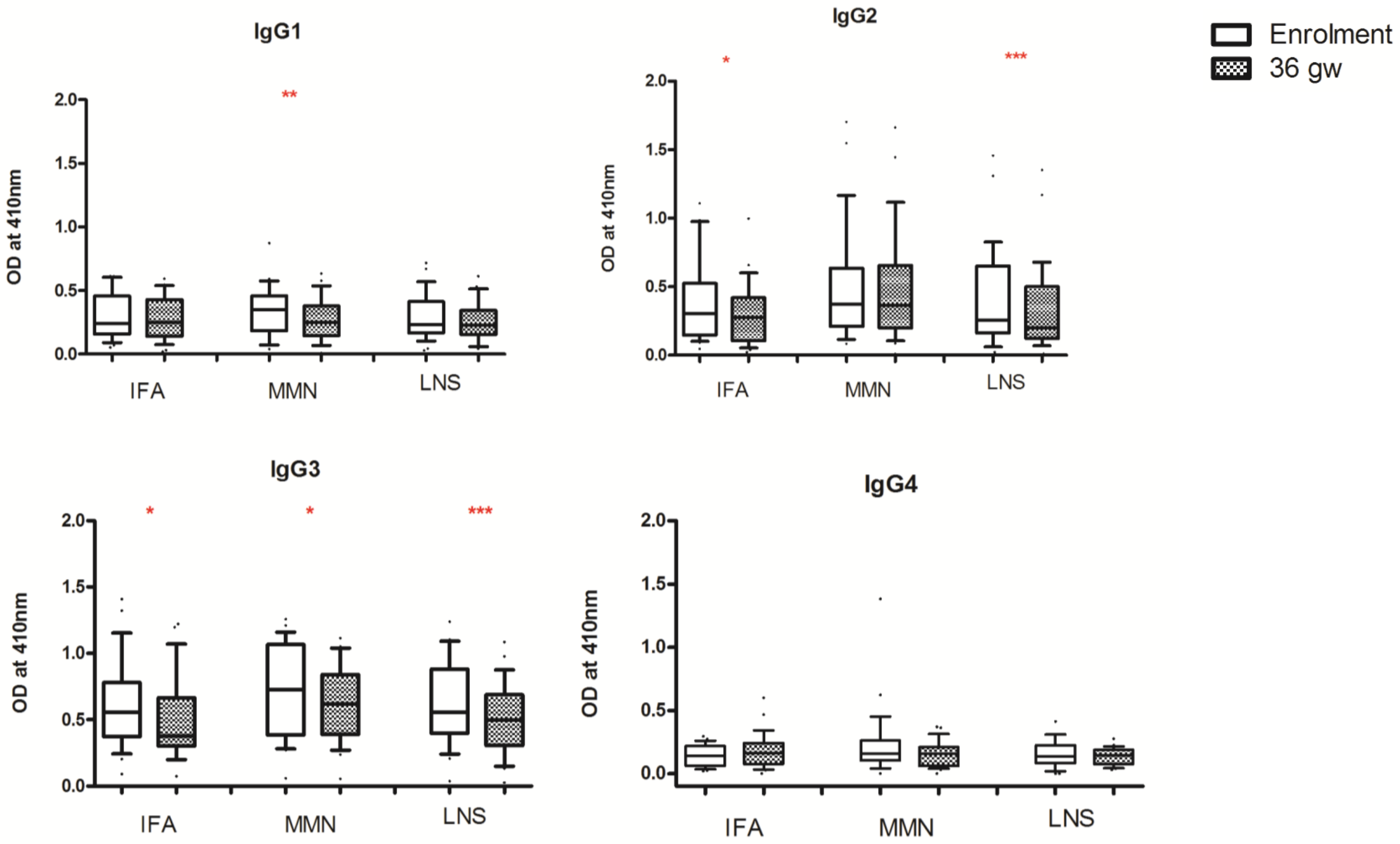

Antibody IgG subclasses presented as optical density (OD) measured at 410nm, categorized by supplementation groups for antigens MSP-1 19kD, MSP-2, MSP-3, PfRh2, EBA-175 and schizont extract (A-F). Kruskal Wallis test performed to determine subclass level differences between supplementation groups (IFA and LNS, MMN and LNS). Wilcoxon matched-pair test was performed for comparing subclass levels between enrolment and 36 gw. No significant differences in subclass levels across the supplementation groups were observed. Subclass levels at enrolment and 36 weeks were significantly different, denoted as $P<0.05$ *, $p<0.005$ **, $p<0.0001$ ***. IFA (n=50), MMN (n=50), LNS (n=50).
